# Guidance on the need for contraception related to use of pharmaceuticals: the Japan Agency for Medical Research and Development Study Group for providing information on the proper use of pharmaceuticals in patients with reproductive potential

**DOI:** 10.1007/s10147-022-02149-1

**Published:** 2022-03-26

**Authors:** Nao Suzuki, Yasushi Takai, Masahito Yonemura, Hiromitsu Negoro, Shinya Motonaga, Noriko Fujishiro, Eishin Nakamura, Seido Takae, Saori Yoshida, Koji Uesugi, Takashi Ohira, Aiko Katsura, Michio Fujiwara, Itsuko Horiguchi, Kenjiro Kosaki, Hiroshi Onodera, Hiroyuki Nishiyama

**Affiliations:** 1 Department of Obstetrics and Gynecology, St. Marianna University School of Medicine, 2-16-1 Sugao, Miyamae-ku, Kawasaki-shi, Kanagawa 216-8511 Japan; 2 Department of Obstetrics and Gynecology, Saitama Medical Center, 1981 Kamoda, Kawagoe-shi, Saitama Japan; 3 Department of Pharmacy, National Cancer Center Hospital East, 6-5-1 Kashiwanoha, Kashiwa-shi, Chiba Japan; 4 Department of Urology, Faculty of Medicine, University of Tsukuba, 1-1-1 Tennodai, Tsukuba-shi, Ibaraki Japan; 5 Pharmacovigilance Section, Office of Clinical Research Support, National Cancer Center Hospital East, 6-5-1 Kashiwanoha, Kashiwa-shi, Chiba Japan; 6 Qol Co., Ltd, 4-3-1 Toranomon, Minato-ku, Tokyo, Japan; 7 Pfizer R&D Japan G.K, 3-22-7 Yoyogi, Shibuya-ku, Tokyo, Japan; 8 Pharmaceutical Research and Manufacturers of America, 3-7-8 Toranomon, Minato-ku, Tokyo, Japan; 9 Takeda Pharmaceutical Company Limited, 2-1-1 Nihonbashi-Honcho, Chuo-ku, Tokyo, Japan; 10 Japan Pharmaceutical Manufacturers Association, 2-3-11 Nihonbashi-Honcho, Chuo-ku, Tokyo, Japan; 11 Novartis Pharma K.K, 1-23-1 Toranomon, Minato-ku, Tokyo, Japan; 12 European Federation of Pharmaceutical Industries and Associations, Japan, 2-1-1 Osaki, Shinagawa-ku, Tokyo, Japan; 13 The Support Center for Clinical Pharmacy Education and Research, Tokyo University of Science, 1-3 Kagurazaka, Shinjuku-ku, Tokyo, Japan; 14 Center for Medical Genetics, Keio University School of Medicine, 35 Shinanomachi, Shinjuku-ku, Tokyo, Japan; 15 Division of Pathology, Center for Biological Safety Research, National Institute of Health Sciences, 3-25-26 Tonomachi, Kawasaki-ku, Kawasaki-shi, Kanagawa 210-9501 Japan

**Keywords:** Proper use of drugs, Anticancer drugs, Oncofertility, Adolescent and young adults, Birth control, Dissemination of information

## Abstract

**Background:**

The U.S. Food and Drug Administration (FDA) and European Medicines Agency (EMA) have published guidelines on the use of cancer treatments in young people of reproductive potential. However, no such guideline is available in Japan. Therefore, this project aimed to gather relevant data and draft a respective guidance paper.

**Methods:**

From April 2019 to March 2021, the Study Group for Providing Information on the Proper Use of Pharmaceuticals in Patients with Reproductive Potential at the Japan Agency for Medical Research and Development gathered opinions from experts in reproductive medicine, toxicology, and drug safety measures. The group considered these opinions, the FDA and EMA guidelines, and relevant Japanese guidelines and prepared a guidance paper, which they sent to 19 related organizations for comment.

**Results:**

By November 2020, the draft guidance paper was completed and sent to the related organizations, 17 of which provided a total of 156 comments. The study group finalized the guidance paper in March 2021.

**Conclusions:**

The “Guidance on the Need for Contraception Related to Use of Pharmaceuticals” (The report of the Study Group for Providing Information on the Proper Use of Pharmaceuticals in Patients with Reproductive Potential, Research on Regulatory Science of Pharmaceuticals and Medical Devices, Japan Agency for Medical Research and Development: JP20mk0101139) is expected to help Japanese healthcare professionals provide fertility-related care and advice to adolescents, and young adults with cancer and their families.

## Introduction

**Fig.1:**
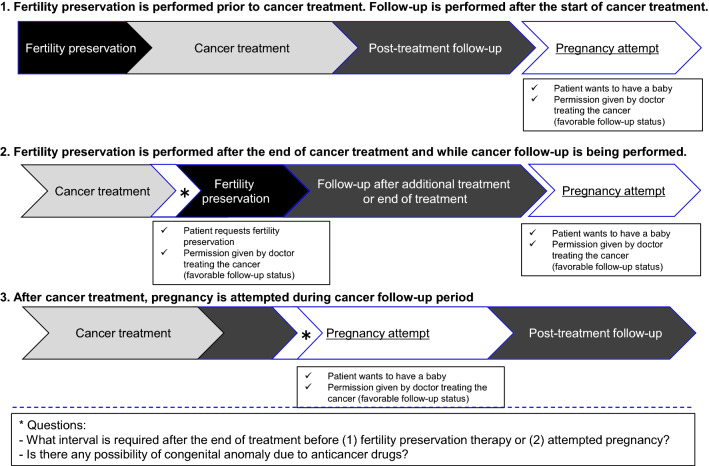
Options for fertility preservation and successful pregnancy in pediatric, adolescent, and young adult cancer patients and associated questions

Cancer survival rates have improved thanks to the advancement of medical care, so it has become more important than ever to provide cancer patients and their family members with information on fertility preservation before starting cancer treatment. Some cancer treatments are known to have gonadal toxicity, so before the start of cancer treatment, fertility preservation options are presented to cancer patients who wish to have children, although cancer treatment is thereby prioritized (see the Fig. 1).

Genetic toxicity means that DNA, chromosomes, or related proteins are affected by exogenous chemical substances, physicochemical factors, or endogenous physiological factors and that the structure and amount of DNA and chromosomes in cells are consequently altered. Unlike other types of toxicity such as hepatotoxicity, neurotoxicity, and carcinogenicity, which can be recognized as symptoms and lesions, genetic toxicity has no visible effects. In somatic cells, toxicity can trigger cancer, and in germ cells, it can cause genetic diseases that will affect the next generation. Therefore, drugs are generally evaluated for genetic toxicity. Occasionally, the interaction between a substance and the DNA is directly measured and evaluated; however, usually other effects are measured, such as the resulting DNA damage, damage response and repair reaction, gene mutations, and chromosomal abnormalities. The range of drugs has increased over the years, so examining the effects of drugs on reproductive processes has grown in importance to ensure that the embryo and the next generation are not affected.

To completely avoid such effects, healthcare professionals must provide patients with appropriate information on measures such as birth control. For this reason, in May 2019 the US Food and Drug Administration (FDA) released the guidance paper “Oncology Pharmaceuticals: Reproductive Toxicity Testing and Labeling Recommendations Guidance for Industry” [1]. The guidance aims to help companies applying to the FDA for approval of a new tumor drug in assessing the reproductive and developmental toxicity of the drug (mainly the impact on embryonic and fetal development) and to minimize the associated potential risks. The guidance specifies the recommendations for package inserts regarding the birth control period after discontinuation of treatment. The concepts it considers are as follows: (1) assessing the effects of various types of drugs on embryo and fetal development; (2) assessing the effects of various types of drugs on embryo and fetal development in specific patient populations; (3) evaluating the necessity of studies on embryo and fetal development for specific purposes, including information such as the results of genetic and general toxicity tests, availability of biomarkers, use of data from recombinant animals; (4) minimizing the risk for embryo and fetal development; and (5) making recommendations on labeling concerning the duration of birth control in male and female patients. In February 2020, the European Medicines Agency (EMA) also published a similar guidance paper, the “Response from SWP [Safety Working Party] to CMDh [Committee for Medicinal Products for Human Use] questions regarding Genotoxicity and Contraception” [2], which aimed to ensure that the health agency and pharmaceutical companies provide clear guidance on the appropriate period of birth control to both healthcare professionals and patients. The EMA document stipulates the recommended birth control period after discontinuing genotoxic drugs in male and female patients and whether these recommendations are applicable to anticancer drugs or to all drugs in general with genotoxic properties.

So far, no guidance on the topic of birth control in cancer patients has been available in Japan. Therefore, the Japan Agency for Medical Research and Development (AMED) Study Group for Providing Information on the Proper Use of Pharmaceuticals in Patients with Reproductive Potential compiled and reviewed opinions from experts in reproductive medicine, toxicology, and pharmaceutical safety measures, reviewed the FDA and EMA guidelines and prepared a Japanese guidance for birth control in patients being treated with anticancer drugs.

## Materials and methods

The AMED Study Group (The report of the Study Group for Providing Information on the Proper Use of Pharmaceuticals in Patients with Reproductive Potential, Research on Regulatory Science of Pharmaceuticals and Medical Devices, Japan Agency for Medical Research and Development; Principal Investigator: Nao Suzuki JP20mk0101139) held 7 times meetings with the mission to prepare within a 2-year period (April 2019 to March 2021) a Japanese guidance similar to the FDA and EMA ones guidance. The aim was to obtain and compile opinions from experts who are familiar with reproductive medicine, toxicology, and drug safety measures and to improve awareness of the proper use of anticancer drugs by publishing a guidance paper on the use birth control during treatment with anticancer drugs.

To prepare the guidance paper, the study group considered various situations and questions that apply to pediatric, adolescent, and young adult cancer patients with reproductive potential. Patients may be offered fertility preservation therapy before the start of cancer treatment, whereby priority is given to cancer treatment (Fig. 1–1). However, fertility preservation may not be an option before cancer treatment, so the group considered the question when patients can start attempting to become pregnant after cancer treatment, i.e., when anticancer drug treatment and radiotherapy will no longer affect gametes and how long patients should wait after the end of treatment to minimize the possibility of congenital anomalies caused by anticancer drugs (Fig. 1–2). A further question, as depicted in Fig. 1–3, was how soon after the end of cancer treatment pediatric, adolescent, and young adult cancer patients can generally have a baby, regardless of whether fertility preservation therapy is performed or not.

Besides considering these questions, the group also referred to the above-mentioned FDA and EMA guidance papers and the following relevant guidelines from Japan: the 2017 Practice Guideline on Fertility Preservation for Pediatric, Adolescent and Young Cancer Patients from the Japanese Society of Clinical Oncology [3, 4] and the 2017 Clinical Guidance on Pregnancy.

The draft Guidance on the Necessity of Birth Control Related to the Administration of Drugs was prepared by November 2020. It was sent to 19 related organization (Supplementary Table), who were asked to provide public comments.

## Results

Seventeen organizations submitted a total of 156 comments, which the study group carefully reviewed. The guidance was finalized in March 2021.

The 2017 Practice Guideline of Fertility Preservation for Pediatric, Adolescent and Young Cancer Patients [3, 4] answers the clinical question of when patients can become pregnant after the end of cancer treatment for each of the seven areas in which cancer occurs, except the female reproductive organs (Table 1). In addition to the information shown in Table 1, the guidance also provides the following information: “After the end of pharmacotherapy, there should be an appropriate wash-out period and birth control period depending on the drug used,” “An appropriate period of birth control should be considered,” and “Birth control should be maintained until anticancer drugs and their metabolites are no longer detected in the body or until a corresponding period of time elapses” [3, 4]. However, the guidance does not specify the optimum time to wait until becoming pregnant after the end of treatment. Instead, the guideline states that “In general, in the case of teratogenic drugs are used, the recommended birth control period is 5 times of the half-life of the drug + 30 days for women and + 90 days for men.” Additional remarks are as follows: “Generally, primordial follicles are less susceptible to the drug, while oocytes may become teratogenic if exposed to drugs. Accordingly, it is desirable to wait a certain interval after the end of chemotherapy in consideration of the time to ovulation of the primordial follicle” and “In male patients, the spermatogenic cells undergo somatic cell division during chemotherapy, which can lead to drug-induced DNA damage, and there is no consensus whether the DNA damage is repaired” [3, 4].

**Table 1:** Information on pregnancy and delivery after cancer treatment described in the 2017 Practice Guideline of Fertility Preservation for Pediatric, Adolescent and Young Cancer Patients (Japanese Society of Clinical Oncology) [3, 4]

Mammary glands CQ4:
If a breast cancer patient wishes to become pregnant, how long should they wait after the end of treatment from the viewpoint of safety, such as teratogenicity related to pharmacotherapy and radiation therapy?
Recommendation: From the viewpoint of teratogenicity of pharmacotherapy, after the drug treatment is completed an appropriate wash-out period and birth control period should be established according to the drugs used. The timing of pregnancy after radiation therapy can be considered based on the risk of recurrence and the pharmacotherapy plan (Recommendation Grade C1)
Urinary system CQ3:
If a patient with urinary cancer wishes to become pregnant, how long should they wait after the end of treatment?
Recommendation: 1. If the patient is male and cryopreserved sperm are available, micro-insemination can be performed whenever the patient wants (Recommendation Grade B). 2. Appropriate periods of birth control should be considered when teratogenic drugs or drugs with insufficient information on fetal safety are used (Recommendation Grade C1)
Pediatric patients CQ4:
What information should be provided regarding pregnancy and delivery after treatment of pediatric cancer patients?
Recommendation: 1. Physicians should explain that if a survivor of pediatric cancer becomes pregnant or the partner of a survivor of pediatric cancer becomes pregnant, there is no significant increase in the risk of congenital anomaly of a newborn associated with cancer treatment. (Recommendation Grade B)
Hematopoietic system CQ6:
What information should be provided regarding pregnancy and delivery after treatment of pediatric cancer patients?
Recommendation: 2. Physicians should explain that it is not clear whether the risk of congenital anomaly in a newborn may increase if a woman becomes pregnant after treatment for hematopoietic malignancies or if the partner of a man who received treatment for hematopoietic malignancies becomes pregnant (Recommendation Grade B)
Bone and soft tissues CQ3:
If a patient with malignant bone and soft tissue tumor wishes to become pregnant, how long should they wait after the end of treatment?
Recommendation: 1. For teratogenic anticancer drugs, birth control should be continued until the anticancer drug or metabolite is no longer detected in the body or the corresponding period elapses (Recommendation Grade C1). 2. In the case of a male patient, if sperm is cryopreserved before anticancer drug treatment or systemic radiotherapy, micro-insemination can be performed at the patient's desired time (Recommendation Grade B)
Brain CQ3:
If a brain tumor patient wishes to become pregnant, how long should they wait after the end of treatment?
Recommendation: 1. For teratogenic anticancer drugs, birth control should be continued until the anticancer drug or metabolite is no longer detected in the body or the corresponding period elapses. (Recommendation Grade C1)
Digestive system CQ4:
If a patient with gastrointestinal cancer wishes to become pregnant, how long should they wait after the treatment is completed?
Recommendation: For teratogenic anticancer drugs, birth control should be continued until the anticancer drug or metabolite is no longer detected in the body or an equivalent period elapses. (Recommendation Grade C1)

**Table 2:** Genotoxic pharmaceuticals: recommendation on duration of contraception after the final dose

Sex	Recommended duration of contraception
Male***	5 × T_1/2_** + 3 months*
Female	5 × T_1/2_** + 6 months*

^*^If the half-life (T_1/2_) is less than 2 days, the duration can be specified as 3 months for men and 6 months for women, irrespective of the length of 5 × T_1/2_

^**^T_1/2_: half-life. The period of 5 × T_1/2_ specified in the above table can be replaced with actual data on elimination of the drug from body, if available

^***^For contraception, the barrier method should be used because it can prevent contact of semen with the vaginal mucosa

The new Guidance on the Need for Contraception Related to Use of Pharmaceuticals defines teratogenicity as an event that inhibits normal embryo or fetal development and may lead to congenital anomalies and uses the term embryo/fetal death to refer to the death of an embryo or fetus regardless of the cause. It refers to the two collectively as developmental toxicity. In addition, it defines genetic toxicity as an action on DNA and chromosomes that induces structural or numerical abnormalities in them, resulting in the onset of cancer and genetic effects in the next generation.

The study group recommends in its guidance suitable types of birth control for reproductive age patients of both sexes and the recommended duration of birth control during and after treatment to minimize the potential risk of developmental and genetic toxicity. Its recommendations are based on discussions about the comments submitted by the 17 organizations and the available Japanese guidelines discussed above. The full text of the guidance is given below.

### Guidance on the need for contraception related to use of pharmaceuticals

#### Introduction

The purpose of this guidance is to minimize the potential risk of developmental toxicity and genotoxicity in the offspring of male and female patients treated with pharmaceuticals and to suggest when and for how long contraception should be recommended during and after treatment. This guidance may be useful for pharmaceutical companies in determining the necessity for recommending contraception in the labeling of pharmaceuticals. Such recommendations should be based on safety information from nonclinical studies, clinical studies, and postmarketing phase. Together with this information, this guidance will also be useful for physicians treating patients in clinical practice.

This guidance reflects current scientific knowledge. As science advances and new information is obtained, the guidance may need to be updated. In addition, this guidance should be read with reference to the relevant guidelines of the International Conference om Harmonization of Technical Requirements for Registration of Pharmaceuticals for Human Use (ICH). Regarding the safety measures to be taken during treatment with any particular pharmaceutical, the treating physician is recommended to consult with the Pharmaceuticals and Medical Device Agency (PMDA), as needed.

In this guidance, the term *teratogenicity* refers to events that may disrupt normal embryo/fetal development and may lead to congenital anomaly, and the term *embryo/fetal lethality* refers to death of the embryo or fetus, irrespective of the cause. *Developmental toxicity* is an inclusive term for both teratogenicity and embryo/fetal lethality. The term *genotoxicity* signifies effects on the DNA or chromosomes, including structural and numerical abnormalities, that eventually cause cancer or genetic flaws in the offspring.

#### Coverage of the guidance

This guidance focuses on information about pharmaceuticals used for prophylaxis or treatment in male and female patients of reproductive potential.

This guidance does not address criteria for the use of contraception in clinical studies. It also does not address gonadal development in children or assisted reproductive technology (such as cryopreservation of gametes).

#### Pharmaceuticals covered

In this guidance, the term *pharmaceuticals* refers to small-molecule pharmaceuticals, biotechnology-derived pharmaceuticals (hereafter, biopharmaceuticals), and related compounds, such as conjugated products. This guidance does not address the reproductive and developmental toxicity risks of radiopharmaceuticals, products for regenerative medicine, vaccines, and so forth. Also, it does not address pharmaceuticals made of biomaterials that have the potential to transmit infection, i.e., that have a risk of transmitting infection to a sexual partner.

### Evaluation of nonclinical studies

An assessment of the effects of pharmaceuticals in the offspring usually refers to the results of reproductive and developmental toxicity studies and genotoxicity studies. When data from these studies are used for risk assessment to ensure safety in humans, adequate consideration must be given to the etiological mechanism of toxic events; the comparison between the exposure level at the no-observed-adverse-effect level (NOAEL) and the clinical exposure level; and variability, such as differences between humans and animals and interindividual differences.

#### Reproductive and developmental toxicity studies

Reproductive and developmental toxicity studies are conducted to obtain information that determines any effects of pharmaceuticals administered to humans on the reproduction and development process. For this purpose, investigations are performed in animals to examine effects on every stage of the reproduction and development process, including effects of exposure to pharmaceuticals on parental animals’ reproductive function, such as gonadogenesis, fertilization, implantation, pregnancy maintenance, delivery, and nursing, and on the offspring, such as fetal lethality, developmental abnormality, growth, and postnatal development. During the studies and in the outcome analysis, potential effects on reproduction and development in humans are assessed in association with pharmacodynamic data and toxicological data. Data on the expected mode of action and level of exposure in humans, on toxicokinetic comparisons, and on the etiological mechanism of reproductive and developmental toxicities are believed to be useful for extrapolating outcomes of animal studies to humans (evaluation of association). The study method used to administer a test substance in a specific stage of the reproduction and development process in animals is chosen to best reflect the exposure of humans to a pharmaceutical and is thought to enable any reproduction and development stages to be identified that will be affected by the test substance.

These nonclinical animal studies are most commonly performed in rats. Three-segment studies, in which the reproduction and development process is divided into 3 segments, are generally recommended. The 3 segments are as follows: study of fertility and early embryonic development to implantation; embryo-fetal developmental toxicity study; and study of pre- and postnatal development, including maternal function. Only the embryo-fetal developmental toxicity study is required to be performed also in nonrodent animals; rabbits are commonly used because, compared with other animal species, the lowest NOAEL of thalidomide was found to be closest in rabbits to that in humans.

For the evaluation method to be used for reproductive and developmental toxicity studies, see ICH Guidance S5 (R3), Guidelines on Detection of Toxicity to Reproductions for Human Pharmaceuticals (PSEHB/PED Notification No. 0129-8 dated January 29, 2021); ICH S6 (R1), Preclinical Safety Evaluation of Biotechnology-derived Pharmaceuticals (PFSB/ELD Notification No. 0323-1 dated March 23, 2012); and ICH S9, Guidelines on Non-clinical Evaluation for Anticancer Pharmaceuticals (PFSB/ELD Notification No. 0604-1 dated June 4, 2010).

#### Genotoxicity study

A genotoxicity study is a study that detects a drug’s potential to cause genetic injury via various mechanisms. It is conducted to detect genetic mutations that develop because of DNA damage and fixation of that damage, such as gene mutations; chromosomal injury, crossover, and numerical changes; and chromosomal morphological or numerical abnormalities. Genotoxicity studies have been used to predict carcinogenicity; however, gene mutations or chromosomal abnormalities of the germ cell line are closely related to hereditary diseases in humans. Accordingly, if an administered substance is suspected to have an effect in the offspring, the clinical significance of its influence on the reproduction and development process is interpreted as being on a par with any associated risks due to cancer.

Most of the chemicals found to be positive in bacterial reverse mutation testing are known to be carcinogenic in rodents. Performing an in vitro test in cultured mammary cells increases the detection sensitivity for carcinogenicity in rodents; however, this test also increases the range of detectable genetic mutations and thus decreases the specificity of the prediction. A single study is incapable of detecting all the effects of genotoxic substances that act via various mechanisms, so genotoxicity must be comprehensively evaluated by conducting multiple studies.

For the evaluation method for genotoxicity, see ICH Guidance S2 (R1), Genotoxicity Testing and Data Interpretation for Pharmaceuticals Intended for Human Use (PFSB/ELD Notification No.0920–2 dated September 20, 2012) and S6 (R1), Preclinical Safety Evaluation of Biotechnology-derived Pharmaceuticals (PFSB/ELD Notification No.0323–1 dated March 23, 2012). For this guidance, the term *genotoxic pharmaceuticals* refer to pharmaceuticals that have been judged to have a genotoxic risk during clinical use, based on the principles of ICH Guidance S2 (R1).

In this guidance, pharmaceuticals that only induce aneuploidy (referred to as aneugenicity) in genotoxicity studies are not categorized as genotoxic pharmaceuticals because induction of aneuploidy is considered to be the result of an effect on the mitotic apparatus, which is composed of proteins, rather than a direct effect on DNA.

### Evaluation of clinical outcomes

In general, at the time of marketing approval of a drug no evidence is available on effects in human offspring because pregnant women are excluded from recruitment into clinical trials. Evidence on the influence of pharmaceuticals on the offspring mostly arises from case reports or the accumulation of national and international reports. However, when interpreting clinical reports on teratogenicity, the causal relationship between the suspected pharmaceutical and the observed adverse event must be evaluated. This evaluation must consider pharmacological and toxicological discussions, as well as the spontaneous incidence rate of congenital anomaly. In addition, when a suspected postmarketing clinical event of a pharmaceutical in a human is available only as a case report, physicians should keep in mind that conclusions on causality are often difficult to draw. Physicians should also consider that the data may not be reliable.

### Considerations on contraception

If nonclinical studies, clinical studies, and/or information from postmarketing phase indicate that a pharmaceutical has a risk of developmental toxicity or genotoxicity, the labeling should include a subsection that describes the need for contraception during and after treatment, suitable types of contraception, and the duration of contraception after discontinuing treatment. Accordingly, it needs to stipulate the need for and duration of contraception in patients and their partners.

As stated above, the scientific background of the description in the labeling is related to multiple factors in the gamete development and maturation process. As described in (“Genotoxic pharmaceuticals”) and (“Nongenotoxic Pharmaceuticals”) below, the aim is to reduce the risk of embryo/fetal developmental toxicity and genotoxicity (i.e., the risks of congenital anomaly and embryo/fetal death). As stated above, the description to be included in the labeling is intended to reduce the pharmaceutical- or parental compound-related risks of embryo/fetal developmental toxicity and genotoxicity; however, those considerations are also applicable to metabolites of concern (for instance, genotoxic metabolites), if applicable.

The toxicological safety of a pharmaceutical is to be evaluated on the basis of studies of fertility and early embryonic development and of pre- and postnatal development, including maternal function, whereby the mechanism of toxicity manifestation, extrapolability to humans, and information from clinical experience are also to be considered. If, on the basis of such considerations, a pharmaceutical is deemed to have a high risk of affecting embryo/fetal development, the labeling should stipulate the necessity for contraception. If the observed effects are limited to effects on gonadal endocrine secretion, gamete production, and fertilization, stipulating a necessity for contraception is thought not to be necessary, although relevant information should still be provided.

If no reproductive and developmental toxicity study of the pharmaceutical of concern has been performed in animals, the pharmaceutical’s possible risk of embryo/fetal developmental toxicity may have to be assessed on the basis of findings of nonclinical and clinical studies, including pharmacodynamic studies, on similar pharmaceuticals. In this case, the recommendations on the necessity of contraception during and after treatment with a pharmaceutical should be based on the respective findings regarding the similar pharmaceuticals.

For a drug conjugate that combines a biopharmaceutical and small-molecule pharmaceutical, the description in the labeling may consider the features of both drugs. For example, an antibody–drug conjugate comprising a combination of genotoxic substances is anticipated to show teratogenicity and cause embryo/fetal death; therefore, the need for contraception and the associated scientific information must be described in the labeling.

In this guidance, the term *duration of contraception* signifies both the recommended duration of contraception intended to avoid fertilization of a gamete exposed to a genotoxic pharmaceutical that leads to pregnancy and the recommended duration of contraception by the barrier method intended to prevent contact of a pharmaceutical transferred into the semen with the vaginal mucosa during sexual activity. If a woman is confirmed to be pregnant during the recommended time span of contraception for herself or her partner, communication of the associated risk and counseling are essential to enable the couple to decide whether or not to continue the pregnancy.

#### *Genotoxic pharmaceuticals* Table 2

##### Contraception in male patients

A number of studies assessed the influence of alkylating agents and genotoxic anticancer agents on spermatogenesis and found that cells undergoing mitosis are vulnerable to genotoxicity [5]. Therefore, the embryo/fetus of the partner may possibly be affected due to DNA damage in a male patient exposed to a genotoxic pharmaceutical. In animal studies, when a male animal administered a genotoxic pharmaceutical was mated with a female animal naïve to that pharmaceutical, effects on the embryo/fetus were observed [6–8]. Therefore, the toxicity risk of genotoxic pharmaceuticals for the embryo/fetus should be minimized by using contraception for the time required for elimination of the drug from the blood after the final dose (five half-lives) plus an additional 3 months. If specific data are available for the pharmaceutical, this period may be replaced by the actual period needed for the pharmaceutical to be eliminated from the blood (this also applies to all respective cases below). The 3-month period covers the period of spermatogenesis and the retention time of nonejaculated sperm [9]. After the above period, newly produced sperm have not been exposed to the genotoxic pharmaceutical and the effect on the embryo/fetus is thought to diminish accordingly. Thus, contraception is not thought to be essential beyond the period specified above.

Even if the partner of a male patient exposed to a genotoxic pharmaceutical uses a contraceptive measure, the pharmaceutical transmitted into the semen can be absorbed through the partner’s vaginal mucosa; consequently, a genotoxicity risk in the partner is conceivable because the partner’s follicles may be exposed to the pharmaceutical during maturation of primordial follicles, as may an embryo/fetus. Therefore, male patients who use a genotoxic pharmaceutical need to use a barrier method to avoid contact of the semen with the vaginal mucosa. If a genotoxic pharmaceutical transmitted into the semen may have come into contact with the partner’s vaginal mucosa, the duration of the partner’s contraception is the same as that for female patients who use a genotoxic pharmaceutical (“Contraception for female patients”).

##### Contraception in female patients

Genotoxic pharmaceuticals may directly affect a patient’s embryo/fetus or potentially cause damage of DNA in oocytes. Most of the follicles that have sustained DNA damage in the granulosa cells or oocytes are suggested to be lost as a result of follicular atresia due to apoptosis [10]. Unlike sperm, oocytes do not undergo cell division or proliferation, and their cell cycle is arrested in the middle of the first stage of the meiosis prophase, which takes place during the fetal stage. Furthermore, if DNA in oocytes is damaged, various genes have been found to be expressed to repair the damage [10]. Thus, genetic mutation is suggested to rarely occur in surviving oocytes, even if they have been exposed to a genotoxic pharmaceutical. The incidence of congenital anomaly in offspring has been reported not to be higher in either cancer survivors treated with genotoxic anticancer agents or atomic bomb survivors [11, 12]. In recent years, in the context of fertility preservation therapy for childhood, adolescent and young adult cancer patients, oocytes and/or ovarian tissues have been harvested even immediately after exposure to genotoxic anticancer pharmaceuticals. In all such cases, a healthy child was born, although the number of cases is limited [13, 14].

Nonetheless, in mice administered a genotoxic pharmaceutical at 1–6 weeks before mating (not after mating), embryo/fetal toxicity events, including miscarriage and fetus malformation, were observed, even though the pharmaceutical was no longer present in the blood at the time of mating [15]. This finding was thought to be attributable to exposure of follicles during the maturation process from primordial follicles (although the study did not investigate whether or not genetic mutations were present in the oocytes). In humans, the time from the start of maturation of primordial follicles until ovulation is reported to take at least about 6 months [16, 17]. If the study result in mice is directly extrapolated to humans, trying to get pregnant or harvesting oocytes/ovarian tissue within 6 months of exposure is potentially associated with a risk for the embryo/fetus.

In conclusion, as a prophylactic measure to avoid exposure of growing follicles to a genotoxic pharmaceutical, the recommended duration of contraception is the period of elimination from the blood after the final dose (five half–lives) plus an additional 6 months.

#### *Nongenotoxic pharmaceuticals *Table 3

##### Contraception in male patients

If a pharmaceutical to be administered to a male patient is confirmed to show developmental toxicity, the related risks that may potentially arise from drug transmission to the partner via the semen must be considered.

If a drug has been transmitted into the semen, the barrier method should be used for contraception until the drug level in semen decreases sufficiently and the partner no longer has a risk of exposure through the vaginal mucosa. If potential developmental toxicity exists because of drug transfer into the sperm or semen, the risk exposure period for the partner can be estimated. However, no evidence is available for determining the duration of contraception in this context. Thus, the time span of drug elimination from the blood after the final dose (five half-lives) is used as a surrogate duration of contraception. However, the drug concentration in the semen does not always correlate with that in the blood. Accordingly, if actual data are available on drug concentration in the semen and drug elimination times, the duration of contraception may be determined on the basis of such data (see Table 3).

**Table 3:** Nongenotoxic pharmaceuticals: recommendation on duration of contraception after the final dose

Sex	Duration of contraception	
	Developmental toxicity: yes	Developmental toxicity: no
Male***	With a risk via transfer to semen: 5 × T_1/2_*,**Without a risk via transfer to semen: not needed	Not needed
Female	5 × T1/2**, ****	Not needed****

^*^This period ensures that the partner no longer has a risk of exposure via transfer to semen is important

^**^T_1/2_: half-life. The period 5 × T_1/2_ specified in the above table may be replaced with data on the actual time required for elimination of the drug from body, if available

^***^For contraception, the barrier method should be used because it can prevent contact with semen

^****^The period should be 5 × T_1/2_ plus 1 month if aneugenicity has been observed. If T_1/2_ is shorter than 2 days, the duration may be set as 1 month, regardless of the length of 5 × T_1/2_

Pharmaceuticals that exhibit only aneugenicity in genotoxicity studies are not categorized as genotoxic pharmaceuticals; however, the same contraceptive measures should be taken as those for pharmaceuticals with potential developmental toxicity.

##### Contraception in female patients

For nongenotoxic pharmaceuticals that have potential developmental toxicity, the duration of contraception should be determined on the basis of the time needed for elimination of the drug from the blood after the final dose (5 × half-life).

Pharmaceuticals that exhibit only aneugenicity in genotoxicity studies may potentially affect meiosis in matured intrafollicular oocytes (which reportedly resumes from 36 h before ovulation in humans), although little evidence is available for an actual increase in fetal chromosome abnormalities. Accordingly, the duration of contraception as a prophylactic measure to avoid exposure can be appropriately determined as the time for drug elimination from the blood after the final dose of the drug (5 × T_1/2_) plus 1 month. In the labeling, the period of 5 × T_1/2_ can be replaced with the actual time needed for the drug to be eliminated from the body.

For pharmaceuticals without developmental toxicity and those for which the risk exposure period can be determined on the basis of the ratio of the largest effective dose to the NOAEL, a toxicity risk for the embryo/fetus can be assumed not to exist; consequently, the duration of contraception does not need to be specified (see Table 3).

### Contraception in specific patient populations

#### Pharmaceuticals for treatment of postmenopausal women

For pharmaceuticals used for treatment of diseases in postmenopausal women, no precautions for contraception need to be described in the labeling.

#### Pharmaceuticals for the treatment of patients younger than 20 years of age

If the target population includes male and female patients of reproductive potential who are younger than 20 years, the same precautions related to contraception should be described in the labeling as for adult patients.

### Description of precautions in the labeling

The labeling of prescription pharmaceuticals should describe the specific duration of contraception on the basis of information on genotoxicity and developmental toxicity obtained from nonclinical studies and information from postmarketing phase in humans. The description should also include the reason for using the pertinent information to justify the selected duration of contraception. If a product has relevant features besides the time required for elimination of the drug from the blood, such features need to be taken into consideration when determining the duration of contraception. Disease characteristics and the duration of treatment also need to be reflected in the information on precautions regarding contraception. Detailed information, including specific contraception methods, should be provided by using material documents, such as interview forms. For medicines requiring pharmacist intervention (PIRM) and over-the-counter drugs, the appropriate contraceptive method to be used as a precaution should also be specified, as is the case for prescription pharmaceuticals.

#### Contraception in male patients

In male patients, not only the effect of a pharmaceutical on the sperm but also the effect of exposure of the man’s partner to the pharmaceutical via the semen should be considered. Therefore, the barrier method must be recommended as a contraception method. If a pharmaceutical produces higher drug concentrations in the semen than in the blood, or is suspected to, the time required for elimination from the semen also needs to be considered.

For pharmaceuticals that are nongenotoxic but have a potential for developmental toxicity or aneugenicity, effects via drug transfer into the sperm or semen need to be considered; however, if available, evidence may be used to determine a specific duration of contraception or to deem contraception unnecessary.

In a male patient whose partner is or may be pregnant or may become pregnant, the following points should be specified in the precautions regarding contraception:

• Specific duration of contraception.

• Necessity of using the barrier method for contraception.

• Evidence data for the precautions regarding contraception, such as the results of genotoxicity studies and reproductive and developmental toxicity studies.

#### Contraception in female patients

In female patients, an appropriate contraception method must be chosen, including use of the barrier method by the partner.

For nongenotoxic pharmaceuticals that have developmental toxicity or aneugenicity potential, it is necessary to consider the NOAEL of developmental toxicity and the effects on the oocytes on the basis of blood concentrations of the pharmaceutical; however, a specific duration of contraception may be determined or deemed unnecessary on the basis of evidence data.

The following points should be specified in the precautions regarding contraception:

• Specific duration of contraception.

• Need to use appropriate contraception.

• Evidence data for the precautions regarding contraception, such as the results of genotoxicity studies and reproductive and developmental toxicity studies.

The differences between this guidance on the necessity for birth control in relation to drug administration and that of the FDA and EMA are presented in Tables 4 and 5.

**Table 4:** The differences between this guidance on the necessity for birth control in relation to drug administration and that of the FDA and EMA- Genotoxic pharmaceuticals: recommendation on duration of contraception after the final dose

	USA	EU	Japan*
Male	5 × T_1/2_ + 3 months	5 × T_1/2_ + 3 months (90 days)	5 × T_1/2_ + 3 months**,^†^
Female	5 × T_1/2_ + 6 months	5 × T_1/2_ + 6 months	5 × T_1/2_ + 6 months^†^
		5 × T_1/2_ + 1 months (for pure aneugenic drugs)	

^*^For pharmaceuticals with short half-lives (T_1/2_ of less than 2 days), PMDA recommends a minimum contraception period of 3 months (for male), and 6 months (for female)

^**^Barrier contraception is suitable for contraception

^†^5xT_1/2_ can be replaced actual data of drug disappearance

**Table 5:** The differences between this guidance on the necessity for birth control in relation to drug administration and that of the FDA and EMA- Nongenotoxic pharmaceuticals: recommendation on duration of contraception after the final dose

	USA	EU	Japan*
Male	Small molecules: 5 × T_1/2_** Biologics: not necessary	Not mentioned	5 × T_1/2_***,^†^
Female	5 × T_1/2_**	Not mentioned	5 × T_1/2_^†^
			5 × T_1/2_ + 1 month****,^†^ (for aneugenic drugs, regardless with/without teratogenicity or embryo-fetal lethality.)

^*^No Teratogenicity and No Embryo-Fetal Lethality: Contraception is not necessary

^**^For pharmaceuticals with short half-lives (5 × T_1/2_ of less than 1 week), FDA recommends a minimum contraception period of 1 week

^***^Barrier contraception is suitable for contraception

^****^For pharmaceuticals with short half-lives (T_1/2_ of less than 2 days), PMDA recommends a minimum contraception period of 1 month

^†^5xT_1/2_ can be replaced actual data of drug disappearance

## Discussion

To avoid the effects of anticancer drugs on children, adolescents, and young adult cancer patients of reproductive potential, the Study Group for Providing Information on the Proper Use of Pharmaceuticals in Patients with Reproductive Potential prepared a Guidance on the Need for Contraception Related to Use of Pharmaceuticals. The guidance is expected to help healthcare professionals provide preconception care to cancer patients of reproductive potential. In addition, the study group hopes that this guidance will be beneficial to children, adolescents, and young adults with cancer, as well as their families. Furthermore, together with the safety information obtained from nonclinical studies, clinical trials, and postmarketing experience, the guidance is expected to help pharmaceutical companies establish recommendations on birth control methods for package inserts and to help healthcare professionals interpret those recommendations.

The guidance describes the basic concepts on the basis of current scientific knowledge and may require revision as new knowledge is obtained and scientific progress is made. In addition, this guidance should be interpretated in light of the relevant International Council for Harmonisation of Technical Requirements for Pharmaceuticals for Human Use (ICH) guidelines. The study group recommends that pharmaceutical companies consult with the Japanese Pharmaceuticals and Medical Devices Agency, as necessary, to ask whether and, if so, what information needs to be provided for individual drugs.
